# Feeling loved versus being loving: perceived partner behavior predicts relationship satisfaction

**DOI:** 10.3389/fpsyg.2026.1773641

**Published:** 2026-02-23

**Authors:** Júlia Halamová

**Affiliations:** Institute of Applied Psychology, Faculty of Social and Economic Sciences, Comenius University in Bratislava, Bratislava, Slovakia

**Keywords:** attachment, attraction, emotion focused therapy, emotionally focused therapy, regard

## Abstract

What matters more for relationship satisfaction, feeling loved or being loving? Johnson’s Emotionally Focused Therapy for couples emphasizes attachment, whereas Greenberg and Goldman’s Emotion-Focused Therapy for couples highlights attraction and regard alongside attachment within a multi-motivational model. The present study addressed a more fundamental question: Does feeling loved predict relationship satisfaction more strongly than feeling loving? Using an extreme groups design comparing clinically distressed (CSI < 51.5; *n* = 213) with highly satisfied individuals (CSI > 78; *n* = 198), we assessed five self-report measures: attraction (IAS), perceived partner attachment behavior (BARE-Partner), perceived own attachment behavior (BARE-Self), perceived partner regard (BLRI: OS), and perceived own regard (BLRI: MO). Results revealed that perceptions of partner behavior consistently predicted satisfaction more strongly than perceptions of own behavior. Effect sizes for partner-perception measures (attachment-partner *d* = 2.64; regard-partner *d* = 2.45) exceeded their self-perception counterparts (attachment-self *d* = 2.32; regard-self *d* = 1.96). Logistic regression confirmed that only partner-perception measures uniquely predicted group membership: attraction (*OR* = 27.58, *p* = 0.003) and perceived partner attachment (*OR* = 4.46, *p* < 0.001), while self-perception measures were non-significant. Notably, the groups differed in which domains were most salient: distressed individuals showed primary deficits in attraction (47%) and feeling valued (35%), whereas satisfied individuals showed primary strengths in attachment security (61%) and attraction (37%). These findings suggest that the subjective experience of feeling loved by one’s partner is more consequential for relationship satisfaction than the perception of being a loving partner, and that different domains may characterize relationship deterioration versus relationship thriving. Clinical implications for assessment and intervention are discussed.

## 1 Introduction

Relationship satisfaction is a cornerstone of individual wellbeing and public health. Satisfied romantic partnerships are associated with better mental and physical health outcomes, longevity, and life satisfaction, while relationship distress predicts depression, anxiety, and reduced quality of life for both partners and their children (Robles et al., 2014). Understanding what predicts relationship satisfaction is therefore not merely an academic question but a matter of significant societal importance.

What predicts relationship satisfaction? Two fundamental questions have shaped the field. First, *which relational domains matter most*—is it attachment security, attraction to one’s partner, or feeling valued and regarded? Second, *whose behavior matters more*—does your perception of how your partner treats you matter more than your perception of how you treat your partner? Despite decades of research, surprisingly little work has directly compared multiple domains or examined the self-versus-partner distinction. The present study addresses both questions simultaneously.

### 1.1 The attachment versus multi-motivational debate

Emotionally Focused Therapy for couples (EFT; Johnson, 2019) and Emotion-Focused Therapy for couples (Greenberg and Goldman, 2008) represent the most empirically supported approaches to couples therapy worldwide (Lebow et al., 2022). However, a fundamental theoretical division exists between these EFT co-founders about the core motivational source of relationship distress.

Johnson (2019) proposes that attachment insecurity lies at the heart of relationship distress. When partners doubt their partner’s accessibility, engagement, and responsiveness, they become trapped in negative interaction cycles—typically pursue-withdraw or attack-defend patterns. From this perspective, rebuilding attachment security is the primary therapeutic goal, with other improvements following naturally. The emphasis is on creating a safe haven and secure base within the relationship (Mikulincer and Shaver, 2016).

Greenberg and Goldman (2008), by contrast, proposed a multi-motivational model identifying three core relational needs: attachment (security and connection), attraction (desire and interest), and identity/regard (feeling valued and respected). From this perspective, couples may struggle primarily with any of these domains—or combinations thereof. A couple might maintain secure attachment but lose attraction, or feel desired but not respected. Therapeutic intervention should therefore target the specific domain(s) that are deficient.

This theoretical debate has significant clinical implications. If attachment is truly primary, therapists should focus on accessibility, responsiveness, and engagement regardless of presenting concerns. If multiple domains contribute independently, assessment should identify which specific needs are unmet, and intervention should be tailored accordingly. Surprisingly, despite the clinical importance of this question, relatively little research has directly compared the relative contributions of attachment, attraction, and regard to relationship satisfaction.

We focus on these two EFT frameworks because they represent the dominant evidence-based approaches to couples therapy and offer competing predictions about relationship dynamics. Other influential models exist, notably Sternberg’s (1986) triangular theory of love (intimacy, passion, commitment), but EFT models are more directly linked to therapeutic intervention and their constructs align closely with available validated measures. Our focus on attachment, attraction, and regard captures the core relational needs emphasized across multiple theoretical traditions while enabling direct clinical application.

### 1.2 Feeling loved versus being loving

A second fundamental question has received even less attention: Within any relational domain, does your perception of your partner’s behavior matter more than your perception of your own behavior? All relationship measures in survey research are fundamentally perceptions—subjective reports of how individuals experience their relationships. Yet these perceptions can be directed toward different targets: one’s own behavior (“I am responsive to my partner”) or one’s partner’s behavior (“My partner is responsive to me”). Both the Brief Accessibility, Responsiveness, and Engagement Scale (BARE; Sandberg et al., 2012) and the Barrett-Lennard Relationship Inventory (BLRI; Barrett-Lennard, 2015) include these bidirectional assessments.

This distinction carries profound theoretical implications. Fromm (1956), in his classic analysis of love, observed that most people focus on being loved rather than on their capacity to love—they ask “Am I lovable?” rather than “Can I love?” While Fromm viewed this as a limitation to overcome, the subjective experience of being loved may be more consequential for wellbeing than the experience of being loving.

This interpretation aligns with Baumeister and Leary’s (1995) belongingness hypothesis, which posits that humans have a fundamental need to feel valued by significant others. Crucially, this theory emphasizes *perceived* belongingness—the subjective sense of being valued—rather than objective relationship status. In romantic relationships, this translates into a potential asymmetry: feeling loved by your partner may matter more than feeling that you love your partner.

Recent large-scale research supports this asymmetry. Joel et al. (2020), in a machine learning analysis of over 11,000 couples, found that perceived partner appreciation was among the strongest predictors of satisfaction. Gordon and Diamond (2023) similarly identified feeling understood and appreciated—perceptions of partner behavior—as critical determinants of relationship quality. These findings converge on a striking conclusion: the phenomenology of feeling loved may be more consequential than the phenomenology of being loving.

### 1.3 Why might partner perceptions dominate?

Several mechanisms could explain why perceptions of partner behavior might predict satisfaction more strongly than perceptions of own behavior. Self-perceptions may be inflated by self-serving biases (Mezulis et al., 2004) most people believe they are good partners, creating a ceiling effect that reduces predictive variance. There may also be a fundamental asymmetry between intentions and impact: you may intend to show love, but what matters for your satisfaction is whether you *perceive* receiving love.

This phenomenological framing also connects to symbolic interactionist perspectives on the self (Cooley, 1902; Mead, 1934). The “looking-glass self” suggests that we experience ourselves through perceived others’ eyes. In romantic relationships, our sense of worth and satisfaction may depend critically on how we perceive our partner sees and treats us—a perception that is fundamentally about the partner’s behavior toward us, not our behavior toward them.

### 1.4 Gaps in the literature

Despite the theoretical importance of both questions, surprisingly little research has addressed them directly. Regarding domains, most studies examine single constructs in isolation—attachment (Mikulincer and Shaver, 2016), communication patterns, or relational beliefs (Gander et al., 2024)—rather than comparing their relative predictive power. A recent *Annual Review of Psychology* synthesis noted that research on attraction in long-term relationships remains disconnected from research on satisfaction (Eastwick et al., 2024). Regarding perspective, the self-versus-partner distinction has rarely been examined systematically.

The present study addresses both questions simultaneously using an extreme groups design (Preacher et al., 2005), comparing clinically distressed individuals with highly satisfied individuals. We employ person-centered analysis to identify which domains and perspectives are most salient for each group, an approach increasingly applied in relationship research (Carlson et al., 2024).

### 1.5 The present study

Using data from 1,090 individuals, we tested two sets of hypotheses:

*Hypothesis 1 (Domains)*: The attachment hypothesis (Johnson’s EFT) predicts that attachment will be the primary discriminator between distressed and satisfied individuals. The multi-motivational hypothesis (Greenberg & Goldman’s EFT) predicts unique contributions from attachment, attraction, and regard.

*Hypothesis 2 (Perspective)*: We hypothesized that partner-perception measures would show stronger discriminative power than self-perception measures, reflecting the primacy of feeling loved over being loving.

## 2 Method

### 2.1 Participants

The sample consisted of 1,090 individuals from 545 heterosexual couples recruited through a Slovak research agency (Go4Insight) using their online panel with quota sampling to ensure demographic representativeness. Participants were compensated with loyalty points redeemable for goods. Data quality was ensured through multiple mechanisms: attention check items, response time monitoring (participants completing the survey in less than 5 min were excluded), and examination for patterned responding. The sample was 49.4% male and 50.6% female, with relationship duration ranging from 1 to 58 years (*M* = 18.4, *SD* = 13.9). The majority were married (78.4%) with the remainder cohabiting (21.6%). Age ranged from 18 to 75 years (*M* = 43.2, *SD* = 13.1). Regarding education, 28.3% had completed secondary education, 45.7% had a university degree, and 26.0% had other educational backgrounds. Inclusion criteria were: (a) age 18 or older, (b) currently in a romantic relationship, and (c) both partners willing to participate. No additional exclusion criteria were applied beyond data quality checks.

### 2.2 Measures

#### 2.2.1 Relationship satisfaction

The Couples Satisfaction Index-16 (CSI-16; Funk and Rogge, 2007).^1^ Assessed global relationship satisfaction (range: 0–81). Individuals scoring below 51.5 were classified as distressed, using the established clinical cutoff (Funk and Rogge, 2007); for the satisfied comparison group, we selected individuals scoring above 78, representing the top quartile of our sample and consistent with extremely high satisfaction levels (approximately 1 *SD* above the mean). Internal consistency was excellent (*α* = 0.98).

#### 2.2.2 Perceived attachment behaviors

The Brief Accessibility, Responsiveness, and Engagement Scale (BARE; Sandberg et al., 2012) measured attachment-related behaviors (12 items). Critically, the BARE includes two subscales: BARE-Self assesses perceptions of one’s own attachment behaviors toward the partner (“I am accessible to my partner”), while BARE-Partner assesses perceptions of the partner’s attachment behaviors toward oneself (“My partner is accessible to me”). Higher scores indicate better perceived attachment functioning. Internal consistency was good (*α* = 0.82 and 0.86, respectively).

#### 2.2.3 Attraction

The Interpersonal Attraction Scale (IAS; McCroskey and McCain, 1974) assessed attraction to the partner across social, physical, and task dimensions (21 items, range: 21–147; higher scores indicate greater attraction). This measure captures the respondent’s attraction toward their partner. Internal consistency was excellent (*α* = 0.94).

#### 2.2.4 Perceived regard

The Level of Regard subscale from the Barrett-Lennard Relationship Inventory (BLRI; Barrett-Lennard, 2015) measured positive regard using two forms: BLRI: MO (Myself-to-Other) assesses perception of one’s own regard toward the partner (“I feel warmly toward my partner”), while BLRI: OS (Other-to-Self) assesses perception of the partner’s regard toward oneself (“My partner feels warmly toward me”). Internal consistency was acceptable (*α* = 0.78 and 0.81, respectively).

### 2.3 Data analysis

We employed an extreme groups design, comparing clinically distressed individuals (CSI < 51.5; *n* = 213) with highly satisfied individuals (CSI > 78; *n* = 198). Extreme groups designs maximize statistical power to detect group differences by comparing individuals at the tails of the distribution (Preacher et al., 2005), though they cannot characterize relationships across the full satisfaction continuum. All analyses were conducted using Python 3.11 with statsmodels (0.14), scipy (1.11), and scikit-learn (1.3) libraries. Data normality was assessed using Shapiro–Wilk tests and visual inspection of Q-Q plots; minor deviations from normality were observed but large sample sizes ensure robustness of parametric tests. There were no missing data for the variables included in the primary analyses. Because our sample consists of couples whose responses may be correlated, the primary logistic regression was conducted using Generalized Estimating Equations (GEE) with an exchangeable correlation structure to account for the non-independence of observations within dyads. This approach provides robust standard errors that properly adjust for the nested data structure (Zeger and Liang, 1986). Analyses included: (1) effect size comparisons (Cohen’s *d*) for all five measures; (2) discriminant function analysis; (3) logistic regression examining unique predictive validity; and (4) primary deficit/strength analysis identifying each individual’s most pronounced area of difference.

## 3 Results

### 3.1 Sample composition

Based on the CSI clinical cutoff, 213 individuals (19.5%) were classified as distressed (CSI *M* = 38.0, *SD* = 12.1). For the satisfied comparison group, 198 individuals scored above 78 (CSI *M* = 80.2, *SD* = 0.8). The total analysis sample was *N* = 411.

### 3.2 Descriptive statistics and intercorrelations

Table 1 presents means, standard deviations, and intercorrelations for all primary study variables in the full sample (*N* = 1,090). All intercorrelations were statistically significant (*p* < 0.001) and moderate to strong in magnitude (*r*s = 0.62–0.81), indicating substantial shared variance among the relationship perception measures. The high correlations between CSI-16 and all predictor variables (*r*s = 0.64–0.79) confirm that these constructs are closely related to relationship satisfaction.

**Table 1:** Descriptive statistics and intercorrelations for primary study variables (*N* = 1,090).

Variable	*M*	*SD*	1	2	3	4	5	6
1. CSI-16	63.51	15.62	—					
2. IAS	125.39	20.21	0.79*	—				
3. BARE-self	24.41	4.21	0.64*	0.64*	—			
4. BARE-partner	24.20	4.45	0.67*	0.66*	0.81*	—		
5. BLRI: MO regard	9.01	3.60	0.67*	0.75*	0.63*	0.62*	—	
6. BLRI: OS regard	7.72	4.44	0.77*	0.77*	0.64*	0.68*	0.73*	—

CSI-16, couples satisfaction index; IAS, interpersonal attraction scale; BARE, brief accessibility, responsiveness, and engagement scale; BLRI: MO, barrett-lennard relationship inventory, myself-to-other form; BLRI: OS, barrett-lennard relationship inventory, other-to-self form. *p < 0.001.

### 3.3 Effect sizes: partner perceptions exceed self perceptions

**Figure 1:**
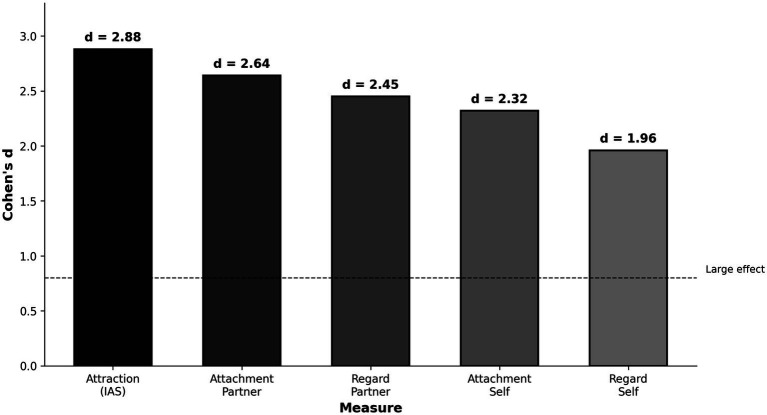
Effect sizes (Cohen’s *d*) for five perception measures. Partner-perception measures (perceived partner attachment, perceived partner regard) show larger effects than self-perception measures (perceived own attachment, perceived own regard).

Table 2 presents descriptive statistics and effect sizes. All measures showed significant group differences (all *p*s < 0.001), but a clear pattern emerged: partner-perception measures consistently showed larger effect sizes than self-perception measures. Attraction showed the largest effect (*d* = 2.88), followed by perceived partner attachment (*d* = 2.64) and perceived partner regard (*d* = 2.45). Self-perception measures showed smaller effects: perceived own attachment (*d* = 2.32) and perceived own regard (*d* = 1.96). See Figure 1.

**Table 2:** Effect sizes comparing distressed and satisfied groups on five perception measures.

Measure	Distressed M (SD)	Satisfied M (SD)	*t*	*d*
Attraction (IAS)	98.5 (18.9)	142.2 (9.8)	29.15***	2.88
Perceived partner attachment	19.6 (3.9)	28.2 (2.5)	26.74***	2.64
Perceived partner regard	1.9 (4.7)	11.0 (2.3)	24.81***	2.45
Perceived own attachment	20.1 (3.8)	28.0 (2.8)	23.49***	2.32
Perceived own regard	5.0 (4.1)	11.4 (2.0)	19.82***	1.96

Distressed: CSI < 51.5 (*n* = 213); Satisfied: CSI > 78 (*n* = 198). ***p < 0.001.

### 3.4 Discriminant function analysis

Discriminant function analysis using all five measures achieved 93.4% classification accuracy (ROC AUC = 0.987). Structure coefficients indicated that attraction (*r* = 0.953) showed the highest correlation with the discriminant function, followed by perceived partner attachment (*r* = 0.925), perceived partner regard (*r* = 0.899), perceived own attachment (*r* = 0.879), and perceived own regard (*r* = 0.812).

### 3.5 Logistic regression: only partner perceptions predict uniquely

GEE logistic regression accounting for dyadic non-independence revealed the study’s most striking finding (Table 3). Within-couple correlation was minimal (*r* = 0.009), indicating negligible dependency between partners’ responses. The full model achieved excellent fit (pseudo *R*^2^ = 0.799). Critically, only partner-perception measures showed significant unique effects. Attraction was the strongest predictor (OR = 27.58, 95% CI [3.00, 253.32], *p* = 0.003): each one standard deviation increase in attraction was associated with approximately 28-fold greater odds of being in the satisfied versus distressed group, followed by perceived partner attachment (OR = 4.46, 95% CI [1.93, 10.31], *p* < 0.001): each one standard deviation increase in perceived partner attachment was associated with 4.5-fold greater odds of satisfaction. Perceived partner regard showed a non-significant effect (OR = 2.49, *p* = 0.199). Neither self-perception measure was significant: perceived own attachment (OR = 1.58, *p* = 0.361) and perceived own regard (OR = 0.51, *p* = 0.387), indicating that changes in perceptions of one’s own behavior were not associated with meaningful changes in odds of group membership when partner perceptions were controlled.

**Table 3:** GEE logistic regression accounting for dyadic non-independence.

Predictor	B	SE	OR	95% CI	*p*
Attraction	3.32	1.13	**27.58**	[3.00, 253.32]	**0.003**
Perceived partner attachment	1.49	0.43	**4.46**	[1.93, 10.31]	**< 0.001**
Perceived partner regard	0.91	0.71	2.49	[0.62, 10.02]	0.199
Perceived own attachment	0.46	0.50	1.58	[0.59, 4.18]	0.361
Perceived own regard	−0.68	0.79	0.51	[0.11, 2.36]	0.387

GEE with exchangeable correlation structure; within-couple correlation = 0.009. Pseudo *R*^2^ (McFadden) = 0.799. Predictors are standardized. Bold indicates significant at *p* < 0.05.

This pattern is remarkable: when all five perceptions are considered simultaneously, only perceptions of partner behavior uniquely predict satisfaction. Perceptions of one’s own loving behavior add nothing beyond perceptions of being loved.

### 3.6 Primary deficit and strength analysis: asymmetric pathways

**Figure 2:**
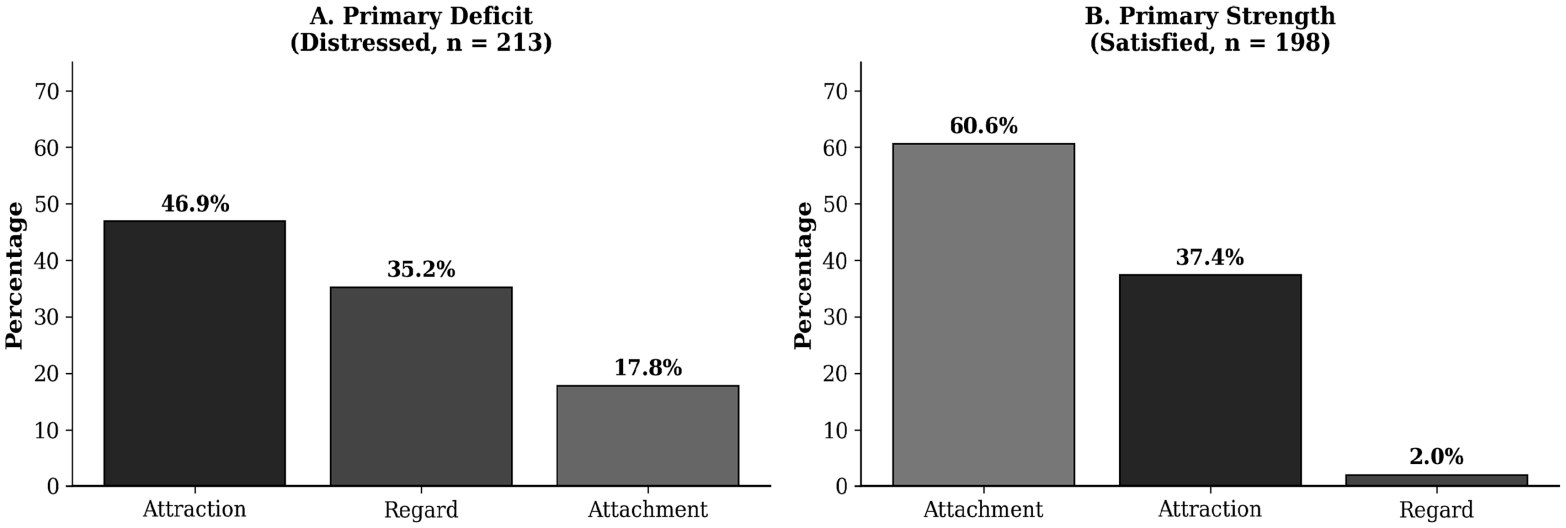
Asymmetric pathways into distress and satisfaction. **(A)** Primary deficits among distressed individuals: attraction and regard dominate. **(B)** Primary strengths among satisfied individuals: attachment dominates. Different domains characterize relationship deterioration versus thriving.

For each individual, we identified which measure showed the largest standardized difference from the comparison group. Results revealed strikingly asymmetric patterns between groups (Figure 2). Among distressed individuals, attraction was the most common primary deficit (46.9%), followed by perceived partner regard (25.8%), perceived partner attachment (15.0%), perceived own regard (9.4%), and perceived own attachment (2.8%). By domain: attraction 46.9%, regard 35.2%, attachment 17.8%.

Among satisfied individuals, the pattern was markedly different. Perceived partner attachment was the most common primary strength (51.0%), followed by attraction (37.4%), perceived own attachment (9.6%), and perceived partner regard (2.0%). By domain: attachment 60.6%, attraction 37.4%, regard 2.0%.

This asymmetry is theoretically significant: distressed individuals are characterized primarily by deficits in attraction and feeling valued (regard), whereas satisfied individuals are characterized primarily by strengths in attachment security and attraction. This suggests that different phenomenological experiences characterize relationship deterioration versus relationship thriving—losing attraction and feeling unvalued may drive couples into distress, while secure attachment may maintain satisfaction.

### 3.7 Self versus partner perceptions

**Figure 3:**
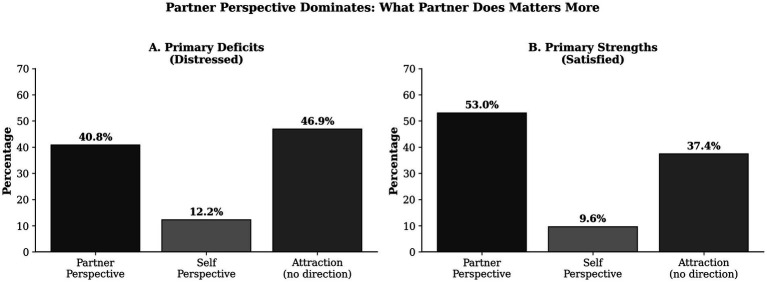
The primacy of feeling loved. **(A)** Primary deficits among distressed individuals: partner-perception measures (40.8%) far exceed self-perception measures (12.2%). **(B)** Primary strengths among satisfied individuals: partner-perception measures (53.0%) far exceed self-perception measures (9.6%). Feeling loved matters more than being loving.

Aggregating across attachment and regard (excluding attraction), we directly compared perceptual targets (Figure 3). Among distressed individuals, 40.8% showed their primary deficit in a partner-perception measure versus only 12.2% in a self-perception measure. Among satisfied individuals, 53.0% showed their primary strength in a partner-perception measure versus 9.6% in a self-perception measure. The partner-to-self ratio was 3.3:1 for deficits and 5.5:1 for strengths.

This striking asymmetry confirms the phenomenological primacy of feeling loved: your perception of how your partner treats you matters substantially more than your perception of how you treat your partner.

## 4 Discussion

The present study addressed two fundamental questions about relationship satisfaction: which domains matter most, and whose behavior matters more. The findings provide clear answers to both. Regarding domains, attraction showed the largest effect size, with attachment and regard also contributing—supporting Greenberg and Goldman’s (2008) multi-motivational model over purely attachment-centric approaches. Regarding perspective, perceptions of partner behavior consistently outperformed perceptions of own behavior, confirming the primacy of feeling loved over being loving.

### 4.1 Hypothesis 1: multiple domains matter

Supporting Greenberg and Goldman’s (2008) multi-motivational model, all three domains distinguished distressed from satisfied individuals with large effect sizes. Attraction showed the largest effect (*d* = 2.88), followed by perceived partner attachment (*d* = 2.64) and perceived partner regard (*d* = 2.45). This pattern challenges purely attachment-centric models while not dismissing attachment entirely—perceived partner attachment was a significant unique predictor (OR = 4.46).

The asymmetric pathways finding adds important nuance. Different domains characterized relationship deterioration versus relationship thriving. Distressed individuals showed primary deficits in attraction (47%) and feeling valued/regarded (35%), with attachment accounting for only 18% of deficits. Satisfied individuals, by contrast, showed primary strengths in attachment security (61%) and attraction (37%), with regard nearly absent (2%).

This asymmetry suggests that losing attraction and feeling unvalued may drive couples into distress, while what keeps couples satisfied is the experience of secure attachment. This pattern has parallels to Herzberg’s (1959) two-factor theory of job satisfaction: regard may function as a “hygiene factor” whose absence causes dissatisfaction, while attachment security may function as a “motivator” whose presence creates satisfaction.

### 4.2 Hypothesis 2: the primacy of feeling loved

The most consequential finding was the consistent superiority of partner-perception measures over self-perception measures. This pattern emerged across effect sizes, structure coefficients, and—most strikingly—in logistic regression, where self-perception measures showed no unique predictive validity whatsoever. When controlling for perceptions of partner behavior, perceptions of own behavior added nothing.

This finding supports and extends Baumeister and Leary’s (1995) belongingness hypothesis. The fundamental human need is not simply to belong, but to *feel* that one belongs—to perceive oneself as valued by significant others. In romantic relationships, this translates into a critical phenomenological asymmetry: the subjective experience of being loved predicts satisfaction; the subjective experience of being loving does not.

Fromm (1956) argued that mature love involves a shift from wanting to be loved to wanting to love. Our data suggest a more nuanced picture: while the capacity to love may reflect psychological maturity, the *experience* of being loved appears more consequential for relationship satisfaction. This is not narcissism—it reflects the fundamental interpersonal nature of romantic wellbeing. We are satisfied in relationships not when we successfully give love, but when we successfully perceive receiving it.

### 4.3 Why self-perceptions may not matter

Several mechanisms may explain why self-perceptions fail to predict satisfaction independently. Self-perceptions of relational behavior may be subject to substantial self-serving bias (Mezulis et al., 2004) most people believe they are good partners, creating restricted variance that limits predictive power. There may also be a fundamental disconnect between perceived intentions and experienced impact: a partner may genuinely perceive themselves as responsive and engaged, yet this self-perception is irrelevant to their own satisfaction. The looking-glass self (Cooley, 1902) suggests that our sense of relational worth comes from how we perceive others see us, not from how we see ourselves.

### 4.4 Clinical implications

These findings suggest fundamental modifications to assessment and intervention. First, clinical assessment should prioritize *perceived partner behavior*. Questions like “Do you feel your partner is emotionally accessible?” and “Do you feel valued by your partner?” may be more diagnostically informative than “Are you emotionally accessible?” or “Do you value your partner?”

Second, interventions should recognize that helping clients feel more loving is insufficient—they must feel more loved. Rather than asking “How can I be a better partner?” each partner should be guided to ask two questions: “How can I help my partner feel more loved?” and “How can my partner help me feel more loved?” This bidirectional framing emphasizes that (a) each partner is responsible for helping the other feel loved, and (b) each partner has legitimate needs that require the other’s involvement to meet.

Third, the asymmetric pathways finding suggests stage-specific interventions. For distressed couples, the priority should be rebuilding attraction and helping partners feel valued again—perhaps through Gottman and Gottman’s (2017) fondness and admiration interventions. For prevention or maintenance, the priority should be sustaining attachment security—perhaps through Johnson’s (2019) emotionally focused interventions that enhance accessibility, responsiveness, and engagement.

Fourth, therapists should help partners communicate their needs explicitly. If feeling loved depends on partner behavior, then partners must know what behaviors make their significant other feel loved. This aligns with Chapman’s (1992) “love languages” concept: partners may be expressing love abundantly but in modalities the recipient does not register.

### 4.5 Limitations and future directions

Several limitations warrant consideration. The cross-sectional design precludes causal inference about whether perception changes precede or follow satisfaction changes. The extreme groups design (Preacher et al., 2005), while maximizing power, excludes middle-range individuals. The Slovak sample limits cultural generalizability.

Our sample consists of couples, and partners’ responses could be correlated. We addressed this by using Generalized Estimating Equations (GEE) with an exchangeable correlation structure to account for dyadic non-independence. The estimated within-couple correlation was minimal (*r* = 0.009), indicating negligible dependency, and the GEE results were virtually identical to standard logistic regression. Nevertheless, future research using Actor-Partner Interdependence Models (APIM; Kenny et al., 2006) could further examine how one partner’s perceptions predict the other partner’s satisfaction.

Additionally, there is potential conceptual overlap between predictor variables and relationship satisfaction. Attraction and feeling valued share definitional space with satisfaction, which may contribute to the observed strong relationships. However, this overlap is theoretically meaningful rather than merely artifactual: the question of which components of the relationship experience most strongly predict global satisfaction remains clinically important even if these constructs are related. The high predictor intercorrelations also raise multicollinearity concerns in the logistic regression. While variance inflation factors were within acceptable ranges (all VIFs < 5), the wide confidence intervals for some predictors suggest some instability, and the claim that self-perceptions “add nothing” may partly reflect shared variance being absorbed by correlated partner-perception measures. The pattern across multiple analytic approaches (effect sizes, discriminant analysis, and logistic regression) provides converging evidence, but future research with independent samples should examine these relationships. A significant limitation is the absence of a general wellbeing or affect measure that could serve as a control variable. Because all predictor variables are relationship-specific, we cannot rule out the possibility that a non-relationship-specific third variable (e.g., positive affect, life satisfaction, depression) accounts for some of the observed associations. Future studies should include such measures to more rigorously establish the unique predictive validity of relationship perceptions beyond general psychological functioning.

Future research should examine these patterns longitudinally. Do changes in perceived partner behavior precede changes in satisfaction, or vice versa? Cross-cultural replication is needed—does the primacy of feeling loved generalize beyond Western samples? Treatment studies should test whether interventions targeting partner perceptions outperform those targeting self-perceptions. Additionally, future studies should employ dyadic analytic approaches (APIM) to examine how one partner’s perceptions predict the other’s satisfaction, and to distinguish actor from partner effects. An intriguing question is whether individual differences in attachment style moderate these patterns—perhaps anxiously attached individuals are particularly sensitive to perceptions of partner behavior, while avoidantly attached individuals show different patterns.

## 5 Conclusion

This study addressed two fundamental questions. First, regarding domains: multiple domains matter, with attraction, attachment, and regard all contributing to relationship satisfaction—supporting multi-motivational over purely attachment-centric models. Different domains characterize distress versus satisfaction, suggesting asymmetric pathways into and out of relationship difficulties. Second, regarding perspective: feeling loved matters more than being loving. When both perceptions are considered, only perceptions of partner behavior predict relationship satisfaction; perceptions of one’s own loving behavior add nothing. Clinical interventions should shift from the self-focused question “How can I be a better partner?” to the interpersonal questions “How can I help my partner feel more loved?” and “How can my partner help me feel more loved?”—and then help couples create the experiences that make feeling loved possible for both partners.

## Data Availability

The raw data supporting the conclusions of this article will be made available by the authors, without undue reservation.
